# C_60_ fullerene and its nanocomplexes with anticancer drugs modulate circulating phagocyte functions and dramatically increase ROS generation in transformed monocytes

**DOI:** 10.1186/s12645-017-0034-0

**Published:** 2018-10-31

**Authors:** Larysa M. Skivka, Svitlana V. Prylutska, Mariia P. Rudyk, Nataliia M. Khranovska, Ievgeniia V. Opeida, Vasyl V. Hurmach, Yuriy I. Prylutskyy, Leonid F. Sukhodub, Uwe Ritter

**Affiliations:** 1 Taras Shevchenko National University of Kyiv, 64 Volodymyrska str., Kiev, 01601 Ukraine; 2 National Cancer Institute, 33/43 Lomonosova str., Kiev, 03022 Ukraine; 3 Sumy State University, 2 Rymskogo-Korsakova str., Sumy, 40007 Ukraine; 4 Institute of Chemistry and Biotechnology, Technical University of Ilmenau, Weimarer str. 25, 98693 Ilmenau, Germany

**Keywords:** C_60_ fullerene, Anticancer drugs, Nanocomplexes, Transformed monocytes, ROS generation, Computer simulation

## Abstract

**Background:**

C_60_ fullerene-based nanoformulations are proposed to have a direct toxic effect on tumor cells. Previous investigations demonstrated that C_60_ fullerene used alone or being conjugated with chemotherapeutic agents possesses a potent anticancer activity. The main aim of this study was to investigate the effect of C_60_ fullerene and its nanocomplexes with anticancer drugs on human phagocyte metabolic profile in vitro.

**Methods:**

Analysis of the metabolic profile of phagocytes exposed to C_60_ fullerene in vitro revealed augmented phagocytic activity and down-regulated reactive nitrogen species generation in these cells. Additionally, cytofluorimetric analysis showed that C_60_ fullerene can exert direct cytotoxic effect on normal and transformed phagocytes through the vigorous induction of intracellular reactive oxygen species generation.

**Results:**

Cytotoxic action as well as the pro-oxidant effect of C_60_ fullerene was more pronounced toward malignant phagocytes. At the same time, C_60_ fullerenes have the ability to down-regulate the pro-oxidant effect of cisplatin on normal cells. These results indicate that C_60_ fullerenes may influence phagocyte metabolism and have both pro-oxidant and antioxidant properties.

**Conclusions:**

The antineoplastic effect of C_60_ fullerene has been observed by direct toxic effect on tumor cells, as well as through the modulation of the functions of effector cells of antitumor immunity.

## Background

Nanocarbon materials attract growing interest as a platform for drug development, including anticancer preparations (Dellinger et al. 2013; Yang et al. 2014; Chen et al. 2015). Fullerene–biomolecule conjugates exhibit a very high antineoplastic efficiency. For example, the chemical attachment of anticancer preparations such as Paclitaxel and Doxorubicin (Dox) to C_60_ fullerene results in an improvement of these drugs’ pharmacokinetics and increases their therapeutic efficacy (Liu et al. 2011; Magoulas et al. 2015; Prylutska et al. 2015). Previous investigations from our research group revealed that pristine C_60_ fullerene possesses a potent anticancer activity per se (Prylutska et al. 2011a, b; Lynchak et al. 2017). In addition, we have demonstrated that conjugation of Dox and Cisplatin (Cis) with the C_60_ fullerene led to significant increase in its toxicity toward various human tumor cell lines in vitro and greatly enhances the inhibitory effect of these drugs on the growth of Lewis lung carcinoma in vivo (Prylutska et al. 2015a, b, 2017 Panchuk et al. 2015). It is well documented that the underlying mechanism of antineoplastic effect of C_60_ fullerene-based nanoformulations is related with the combination of the direct effect on tumor cells (modulation of oxidative stress, apoptosis, necrosis, and autophagy), their effect on tumor microenvironment (reduction of the blood supply to tumor tissues), and activation of the host immune system (Dellinger et al. 2013; Harhaji et al. 2007; Shi and Li 2012).

There are limited and controversial data concerning immunomodulatory effects of C_60_ fullerenes. Liu et al. (2009) have shown that C_60_ fullerene and its derivatives exert potent immunomodulatory pro-inflammatory activities: increase TNFα production and enhance cell immune response, and show almost no adverse effect to the viability of immune cells. Fujita et al. (2009) revealed that genes involved in the inflammatory response, oxidative stress, metalloendopeptidase activity, as well as MHC expression were upregulated in immune cells after the exposure to C_60_ fullerene. Zogovich et al. (2009) have reported a significant increase in splenocyte production of the immunoregulatory free radical nitric oxide (NO) after the treatment with C_60_ fullerene. A special attention in the analysis of C_60_ fullerene immunomodulatory properties is given to their influence on phagocytes.

Phagocytes are credited with a crucial role in the development of the immune response. Professional phagocytes (monocytes, dendritic cells, macrophages, neutrophils, and mast cells) constitute the first line of cellular immune defense. These cells are important players in the antitumor protection and are the link between the innate and adaptive immunity (Murray and Wynn 2011; Galdiero et al. 2013; Sica et al. 2015). Monocytes/macrophages and neutrophils activate, orient, and regulate adaptive responses. These cells are characterized by high plasticity and can possess different metabolic profiles, depending on the activating stimuli. There are two main diametrally opposite polarizational state of macrophages/neutrophils (not excepting numerous intermediate metabolic states): classical (M1 and N1, respectively) and alternative (M2 and N2, respectively). The main distinctive features of classically polarized phagocytes are the generations of effector molecules (reactive oxygen and nitrogen species) and cytokines (TNFα, IL1β, IL6, etc.) participating in the inflammation and promotion of Th1 immune response. These cells mediate immune defense against intracellular pathogens and malignant tumors. Characteristic features of alternatively polarized phagocytes are their participations in the resolution of inflammation, tissue repair, and switching of adaptive immune response to Th2 profile. These cells express high levels of receptors involved in endocytosis, produce anti-inflammatory cytokines, and metabolize arginine to ornithine and polyamines. Alternatively, polarized phagocytes can possess immunosuppressive activity, participate in tissue remodeling, and promote tumor growth and angiogenesis (Locati et al. 2013; Okabe and Medzhitov 2016; Egners et al. 2016).

Regardless of the route of C_60_ fullerene administration chosen, phagocytes are the first cells of the body immune system, which are influenced by these nanoformulations. Therefore, a greater understanding of interactions between C_60_ fullerene and phagocyte will enable the creation of better predictive models of their therapeutic efficacy as well as adverse outcomes following exposure. Russ et al. (2016) suggested that C_60_ fullerene’s effect on phagocyte signaling is achieved through endocytosis/pinocytosis as well as passive diffusion of this nanostructure. According to the limited literature data available, the character of the C_60_ fullerene’s influence on phagocytes depends on their geometric structure, dose, duration of exposure, and the initial functional state of the cells. C_60_ fullerene and their derivatives at low concentrations exerted mainly negative effect on the functions of nonsensitized macrophages in vitro by inhibiting the myeloperoxidase activity, and suppressed the expression of CD54 involved in the adhesion (Vesnina et al. 2011; Pirutin et al. 2012). Inhibitory effect of C_60_ fullerene on pro-inflammatory (classically)-activated macrophages was described in rats with experimental adjuvant-induced arthritis (Vesnina et al. 2012). Many authors are unison in their opinions that C_60_ fullerenes dramatically affect the phagocyte oxidative metabolism and can even induce apoptosis of macrophage by changing the mitochondrial membrane potential (Santos et al. 2014; Zhang et al. 2015; Yu et al. 2015). Based on our previous experience, in vivo anticancer effects of C_60_ fullerene and C_60_ + Dox nanocomplexes are accompanied by the increase of macrophage oxygen-dependent cytotoxicity toward authologic tumor cells (Prylutska et al. 2015). In addition, our previous results revealed direct toxic effects of C_60_ fullerene and its nanocomplexes with anticancer drugs on transformed leukocytes including myelocytic cells (Panchuk et al. 2015; Scharff et al. 2008; Franskevych et al. 2015).

The main aim of this study was to investigate the effect of C_60_ fullerene and its nanocomplexes with anticancer drugs on human phagocyte metabolic profile in vitro. Potential mechanisms of this effect have been analyzed using docking experiments of direct interactions between the C_60_ fullerene and phagocyte pattern-recognition receptors as well as cytochrome p450 (CYP). A study of the effect of C_60_ fullerene and its nanocomplexes with anticancer preparations on pro-monocytic, human myeloid leukemia cell line U937 was also performed to explore the potential application of C_60_ fullerene in the therapy of acute myelocytic leukemia.

## Methods

### Preparation of C_60_ fullerene aqueous colloid solution

The pristine C_60_ fullerene aqueous colloid solution (C_60_FAS; final concentration 0.15 mg/ml) used in the experiments was prepared according to the protocols developed previously (Scharff et al. 2004; Prylutskyy et al. 2014; Ritter et al. 2015).

### Preparation of C_60_ + Cis and C_60_ + Dox nanocomplexes

Cis solution (Cisplatin-TEVA, Pharmachemie B.V., 0.5 mg/ml) was immobilized on the C_60_ fullerene according to the protocol developed by our research group (Prylutskyy et al. 2015). The initial solution of C_60_FAS (final concentration 0.15 mg/ml) and Cis (final concentration 0.15 mg/ml) was mixed in 1:1 volume ratio. Afterward, the mixture was treated for 20 min in ultrasonic disperser and, next, subjected to overnight magnetic stirring at the room temperature.

Dox (Doxorubicin-TEVA, Pharmachemie B.V., lyophilized powder, 10 mg) was dissolved in saline to obtain a final concentration of 0.15 mg/ml. It was immobilized on the C_60_ fullerene according to a previously described protocol (Prylutskyy et al. 2014, 2015). Specifically, C_60_FAS (final concentration 0.15 mg/ml) and Dox (final concentration 0.15 mg/ml) were mixed in 1:2 volume ratio, and the resulting mixture was treated for 20 min in the ultrasonic disperser, and then, it was subjected to overnight magnetic stirring at the room temperature.

### Simulation

The geometric structures for CYP (3tis—crystal structure of the complex between human CYP 3A4 and desthiazolylmethyloxycarbonyl ritonavir) and Toll-like receptors (TLRs) (2z7x—crystal structure of the TLR1–TLR2 heterodimer, Homo sapiens, Eptatretus burger; 4g8a—crystal structure of human TLR4 polymorphic variants, D299G and T399I) from the PDB base were used in calculations.

Computational methods such as docking and molecular dynamics (MD) simulations were applied. We used an algorithm of systematic docking (SDOCK+) implemented in QXP docking software, which has shown a higher reproducibility of compound conformation with a minimum Root mean square deviation (Rmsd) in comparison with the crystallographic data (Warren et al. 2006). The maximum number of SDOCK+ routine steps was set to 200, and the 10 best protein (target)-C_60_ fullerene complexes, based on built-in QXP scoring function (McMartin and Bohacek 1997), were retained for each compound. The optimal position of obtained complexes was selected based on their overall energy.

Molecular dynamics simulation was performed for up to 25 ps to evaluate the stability of protein (target)–C_60_ fullerene complexes. For the calculation, we used NPA algorithm (Sturgeon and Laird 2000). The above method is one of the most accurate and sensitive methods, and it generates true ensemble trajectories (Bond et al. 1999). Within the calculation, the next main parameters used were temperature (in K)—300; pressure (in kPa)—100; water molecules treated as rigid bodies.

### Cell isolation

Four healthy adult men aged 21 ± 2 years were recruited to participate in the study. Approval was obtained from the ethical committee of Taras Shevchenko National University of Kyiv, and informed consent was obtained from all subjects before the commencement of the study.

Fresh blood (20 ml) was obtained from volunteers and mixed with 50 μl of preservative-free heparin (Hospira, UK). Sterile dextran was added to a final concentration of 0.6%, and the cells were allowed to settle at 37 °C until the red cells had sedimented. The buffy coat was then removed, washed in Hanks’ buffered salt solution, and then resuspended in RPMI 1640 medium containing 20% fetal calf serum(FCS), penicillin (50 U/ml), and streptomycin (50 μg/mL) (Evans et al. 1996).

### Cell line

Human myeloid lineage cells U937 were kindly supplied by the Bank of Cell Cultures and Transplantable Experimental Tumors of R.E. Kavetsky Institute of Experimental Pathology, Oncology and Radiobiology of NAS of Ukraine (Kyiv, Ukraine). Cells were cultured in vitro in Dulbecco-modified Eagle medium (DMEM, Sigma, USA) supplemented with 10% fetal calf serum (FCS), penicillin (100 U/ml), and streptomycin (100 μg/ml) at 37 °C in 5% CO_2_.

### Cell incubation with C_60_ fullerene, Cis, Dox, and their nanocomplexes

Prior to the metabolism assays, 200 µl of cell suspension in RPMI 1640 medium or 200 µl of heparinized whole blood was treated with C_60_ fullerene (final concentration 0.15 mg/ml), Cis (final concentration 0.15 mg/ml), Dox (final concentration 0.15 mg/ml), and nanocomplex of C_60_ fullerene with Cis (mixed in 1:1 volume ratio) or Dox (mixed in 1:2 volume ratio) for 30 min.

### Nitrite assay

Nitrite-level determination was performed to evaluate NO release into the conditioned media of human peripheral blood monocytes and granulocytes as described earlier (Neil 2009; Skivka et al. 2013). In brief, after 24 h of cultivation, the culture supernatants were collected, and the nitrite concentration in each supernatant was assayed by the Griess reaction. Equal volumes of 2% sulfanilamide in 10% phosphoric acid and 0.2% naphthylethylene diamine dihydrochloride were mixed to prepare the Griess reagent. The reagent (100 μl) was added to equal volumes of the supernatant, and the mixture was then incubated for 30 min at room temperature in the dark. The A550 of the formed chromophore was measured using a plate reader. The nitrite content was calculated with sodium nitrite as a standard. Each sample was assayed for nitrite in triplicate. Each value was divided by the number of viable cells and expressed as nitrite level per 10^6^ cells. The mean value and SD were calculated with normalized values.

### Intracellular ROS assay

Reactive oxygen species (ROS) levels were measured using 2′7′-dichlorodihydro-fluorescein diacetate (carboxy-H2DCFDA, Invitrogen) as previously described (Skivka et al. 2013). In brief, heparinized whole blood was incubated with PBS containing 10 μM carboxy-H2DCFDA for 30 min at 37 °C to measure ROS production by peripheral blood monocytes and granulocytes. A short recovery time was allowed for the cellular esterases to hydrolyze the acetoxymethyl ester or acetate groups and render the dye responsive to oxidation. Erythrocytes were lysed with lysis buffer. The cells were then transferred to polystyrene tubes with cell-strainer caps (Falcon, Becton–Dickinson, USA) and analyzed with flow cytometry (excitation: 488 nm; emission: 525 nm). Only living cells, gated according to scatter parameters, were used for the analysis. Granulocytes or monocytes were gated according to forward and side scatters. Phorbol 12-myristate 13-acetate (PMA) (Sigma-Aldrich) was used to evaluate nonspecific reactivity reserve in phagocytes (Shapiro et al. 2011; Skivka et al. 2015). Reactivity reserve was characterized by the modulation coefficient (MC) that was calculated by the following formula:

$${\text{MC }} = \left( {\left( {S - B} \right)/B \times 100} \right),$$

where *S* is the ROS value in probes stimulated with PMA in vitro, and *B* is ROS value in unstimulated probes (basal value).

### Phagocytosis assay

The flow cytometry phagocytosis assay was performed as previously described (Skivka et al. 2013). In brief, the fluorescein isothiocyanate(FITC)-labeled heat-inactivated *Staphylococcus aureus* Cowan I bacteria (collected by the Department of Microbiology and General Immunology of Taras Shevchenko National University of Kyiv) at the concentration of 1 × 10^7^ cells/ml in the volume of 5 μl were added to heparinized whole blood. All samples were incubated at 37 °C for 30 min. At the end of the assay, phagocytosis was arrested by the addition of cold stop solution (PBS with 0.02% EDTA and 0.04% paraformaldehyde). Erythrocytes were lysed with lysis buffer. Results were assessed using FACSCalibur flow cytometer and CellQuest software (Becton–Dickinson, USA). Granulocytes or monocytes were gated according to forward and side scatters. The results were registered as the percentage of cells emitting fluorescence after a defined culture period (phagocytosis percentage, PP) and as phagocytosis index (PI) that represents the mean fluorescence per one phagocytic cell (engulfed bacteria by one cell).

### Determination of arginase activity

Arginase activity was measured in cell lysates by standard colorimetric method with some modifications (Skivka et al. 2013). In brief, 100 μl of 0.1% Triton X-100 and 100 μl of 50 mMTris-HCl (pH 7.5), containing 10 mM MnCl_2_, were sequentially added to cell samples. Phagocyte arginase was then activated by heating of the mixture at 56 °C for 7 min. The reaction of l-arginine hydrolysis by arginase was carried out by incubation of the mixture containing activated arginase, with 100 μl of l-arginine (0.5 M; pH 9.7) at 37 °C for 2 h, and was stopped by the addition of 800 μl of the mixture of acidic solution (H_2_SO_4_:H_3_PO_4_:H_2_O = 1:3:7). For colorimetric determination of urea, α-isonitrosopropiophenone (40 μl, 9% solution in ethanol) was added, and the mixture was incubated at 95 °C for 30 min and then at 4 °C for 30 min. The urea concentration was determined spectrophotometrically at 540 nm with the use of a microplate reader. Each condition was tested in triplicate and the experiments were repeated at least three times. Each value was divided by the number of viable cells and expressed as urea level/h per 10^6^ cells. The mean value and SD were calculated with normalized values.

### Cell death assay

Apoptosis was assessed by staining cells with Annexin V-FITC and counterstaining with propidium iodide (PI) with the use Annexin V-FITC Apoptosis Detection Kit (DojindoEUGmbH, Munich, Germany) according to the manufacturer’s instructions. In brief, 2 × 10^5^ cells were placed into wells of a 96-well flat-bottomed plate and were either treated with C_60_ fullerene, Cis, Dox and their nanocomplexes at maximum concentration (0.15 mg/ml) for 24 h. Untreated cells were used as a control. Afterward cells were washed twice with PBS and stained with 5 μl Annexin V-FITC and 5 μl PI in binding buffer for 10 min at room temperature in the dark. Cells from each sample were then analyzed by FacsCalibur flow cytometer (BD Biosciences). The data were analyzed using CELLQuest software (BD). PI detects cells that have lost CPM integrity (i.e., necrotic and secondary necrotic cells), whereas Annexin V detects early apoptotic cells.

### Statistical analysis

All experimental results are reported as mean ± SD. Statistical significance of the results was determined by *t* test (unpaired, two-tailed) and the nonparametric Mann–Whitney test, comparing two groups of independent samples. Means were compared, and differences were considered significant at *p* values of 0.05 or less.

## Results and discussion

Phagocytes play a crucial role in antitumor immunity. Modulation of their metabolism is involved in anticancer effect of conventional anticancer preparations (Banciu et al. 2008; Wong et al. 2014) and can be considered as one of the mechanisms of C_60_ fullerene antineoplastic action. C_60_ fullerene can affect phagocyte metabolism by activation of membrane receptor followed (or no) by the endocytosis and/or by interaction with intracellular receptive structures after passive diffusion. Entry pathways of C_60_ fullerene-based nanoformulations shift from passive diffusion to receptor-mediated endocytosis with increasing particle size (Russ et al. 2016; Zhang et al. 2011). As we reported previously (Prylutskyy et al. 2013, 2014; Ritter et al. 2015), the probe microscopy revealed in our preparations randomly arranged individual C_60_ molecules with a diameter of ~ 0.7 nm and their bulk sphere-like aggregates with a height of 2–100 nm in C_60_FAS. Therefore, C_60_ fullerenes and their nanocomplexes with anticancer drugs can exert additive membrane-dependent and direct intracellular effect on phagocyte metabolism. It was important to investigate the effect of C_60_ fullerenes and their nanocomplexes with anticancer drugs on different metabolic reactions of nonsensitized human peripheral blood phagocytes as well as myeloid leukemia cells U937, and revealed potential receptive membrane and intracellular structures, which could be involved in such effect.

### Molecular docking analysis

The molecular docking approach allows simulating the behavior of small molecules such as C_60_ fullerenes and their nanocomplexes with anticancer drugs in the binding sites of target cells. It also gives us an opportunity to predict the effect of such small molecules on metabolic processes in cells (Hurmach et al. 2014; Guedes et al. 2014).

Majority of metabolic phagocyte functions depends on the activation of pattern-recognition receptors, among which TLRs are most important. TLR signaling is involved in activation/regulation such fundamental phagocyte functions as phagocytosis, oxygen-dependent and oxygen independent cytotoxicity, arginine metabolism, antigen presentation, cytokine synthesis etc. (Parker et al. 2005; McCoy and O’Neill 2008). Some TLRs are considered as a binding site for C_60_ fullerene (Turabekova et al. 2014). Taking into account the profound influence of C_60_ fullerene-based nanoformulations on cell oxidative metabolism, one of its significant intracellular targets can be the CYP—a key component of the monooxygenase system. Unlike other hemoproteins having in cell usually one activity and well-defined function, CYP alongside monooxygenase activity can exhibit oxidase one, generating such ROS as superoxide and hydroxyl radicals, hydrogen peroxide. Poor coupling of the CYP catalytic cycles results in continuous ROS generation. CYP is reported to produce 12(S)-hydroxyheptadeca-5Z,8E,10E-trienoic acid (12-HHT) that is overexpressed in classically activated macrophages (Zangar et al. 2004; Frömel et al. 2013).

### Cytochrome p450

**Fig. 1:**
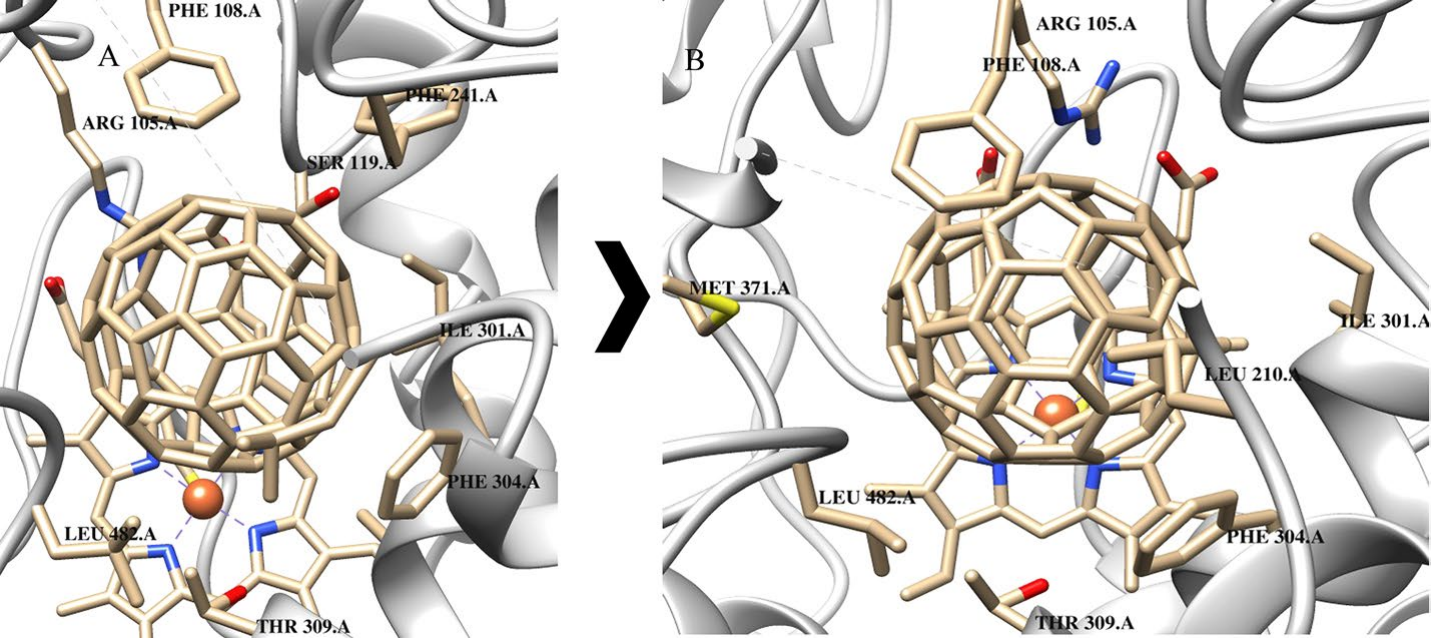
Model of C_60_ fullerene-3tis binding: **A** docking, **B** MD simulation

The active site for the majority of the monoxidation reactions, which CYP enzymes catalyze, contains a heme group during the catalytic processes (Meunier et al. 2004). It is known that CYP3A4 can accommodate multiple substrates owing to the large size of the active site [(500–1532) Å] (Cui et al. 2013). So, in both cases C_60_ fullerene can fill in the binding site, interact with a large number of amino acids and create cation π-system with cofactor. For example, in the case of PDB 3tis structure, C_60_ fullerene involved in the stacking interaction with Phe 304 and Phe 108 (Fig. 1).

The docking and MD results indicate that C_60_ fullerene forms stable complex with CYP: the respective free energy of complexation is − 54.4 kJ/mol after docking and − 47.5 kJ/mol after MD simulation. The energy of steric clashes for C_60_ fullerene is higher after MD simulation than that after docking: it means that C_60_ fullerene–CYP complex after MD simulation is more rigid, despite that its overall energy is less (18.8 kJ/mol after docking and 25.7 kJ/mol after MD simulation). One can assume that this is due to changes in the binding site. Furthermore, the distance between cofactor and C_60_ fullerene decreased: it equals to 5.4 Å after docking and 4.52 Å after MD simulation. At the same time, the Rmsd value for C_60_ fullerene is not significant: it equals to 0.56 Å (in turn the Rmsd value of protein is 2.53 Å). As a result C_60_ fullerene deepens into the binding site and gets stuck among some amino acids (e.g., Phe 108 and Phe 304 (Fig. 1); Rmsd values are 3.5 and 1.2 Å, respectively).

### TLRs

**Fig. 2:**
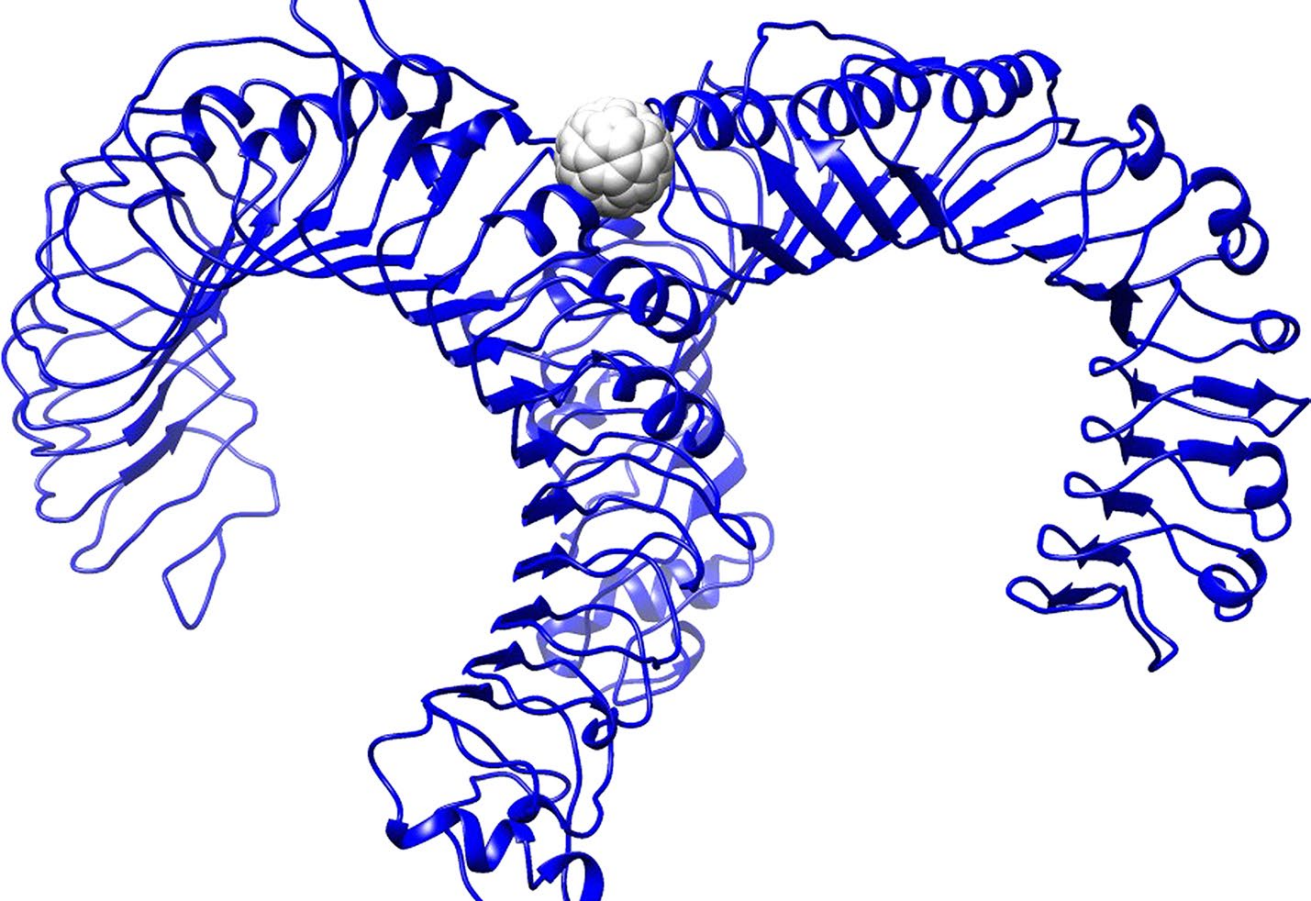
C_60_ fullerene–TLR1/TLR2/ECD-bound structure

**Fig. 3:**
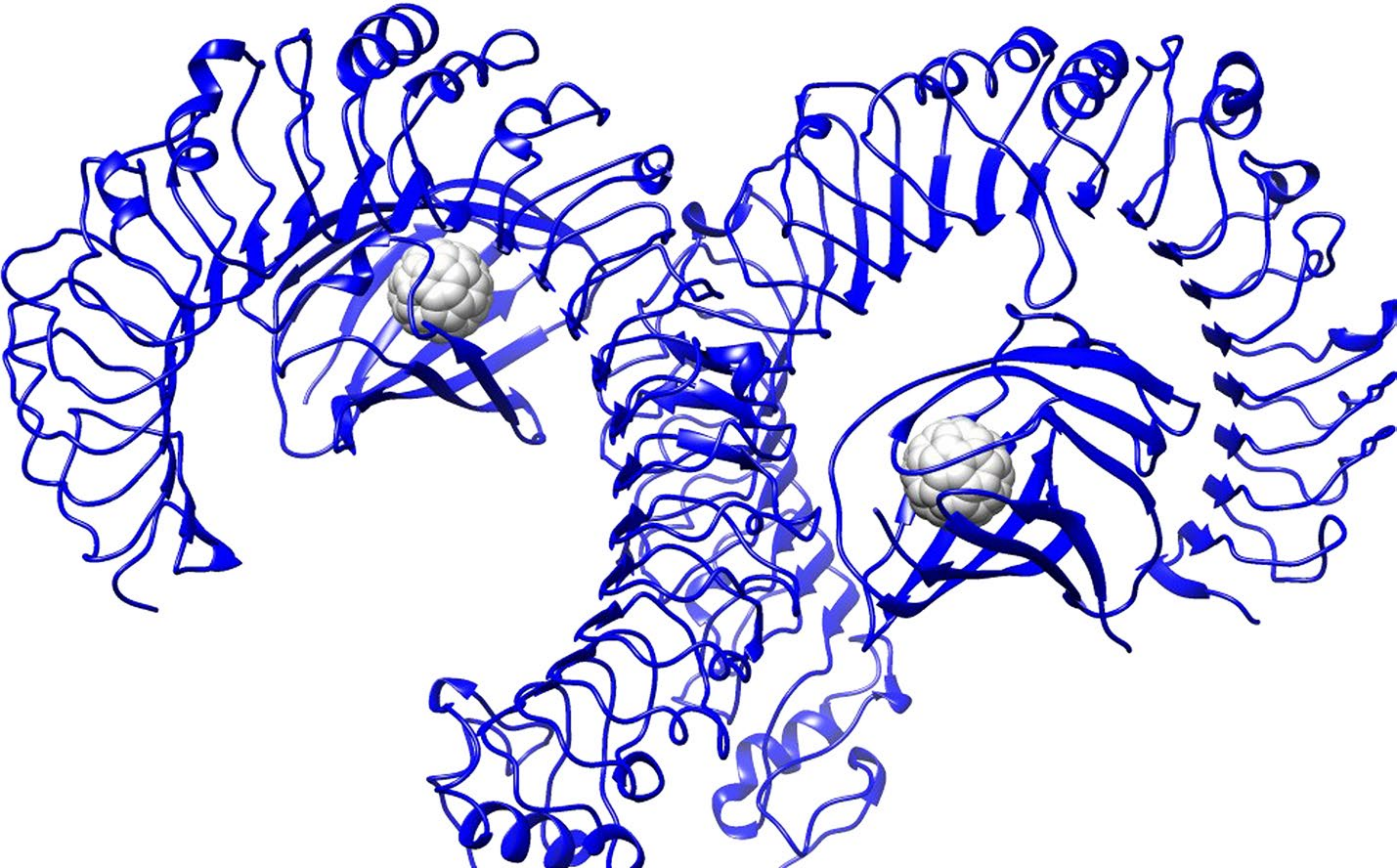
C_60_ fullerene–TLR4/MD-2-bound structure

Based on previous data (Turabekova et al. 2014), docking and MD simulation were carried out using TLR1/TLR2 [Fig. 2; we investigated the binding of C_60_ fullerene in extracellular domains (ECDs)] and TLR4 (Fig. 3; we investigated the binding of C_60_ fullerene in MD2 domain).

**Fig. 4:**
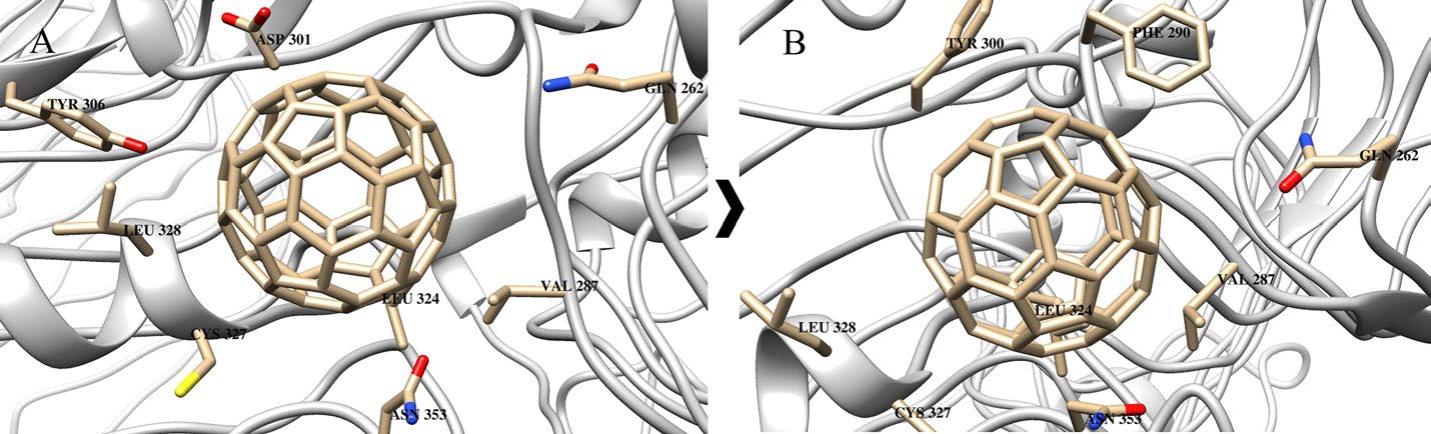
C_60_ fullerene–TLR1/TLR2/ECD-bound structure: **A** docking, **B** MD simulation

**Fig. 5:**
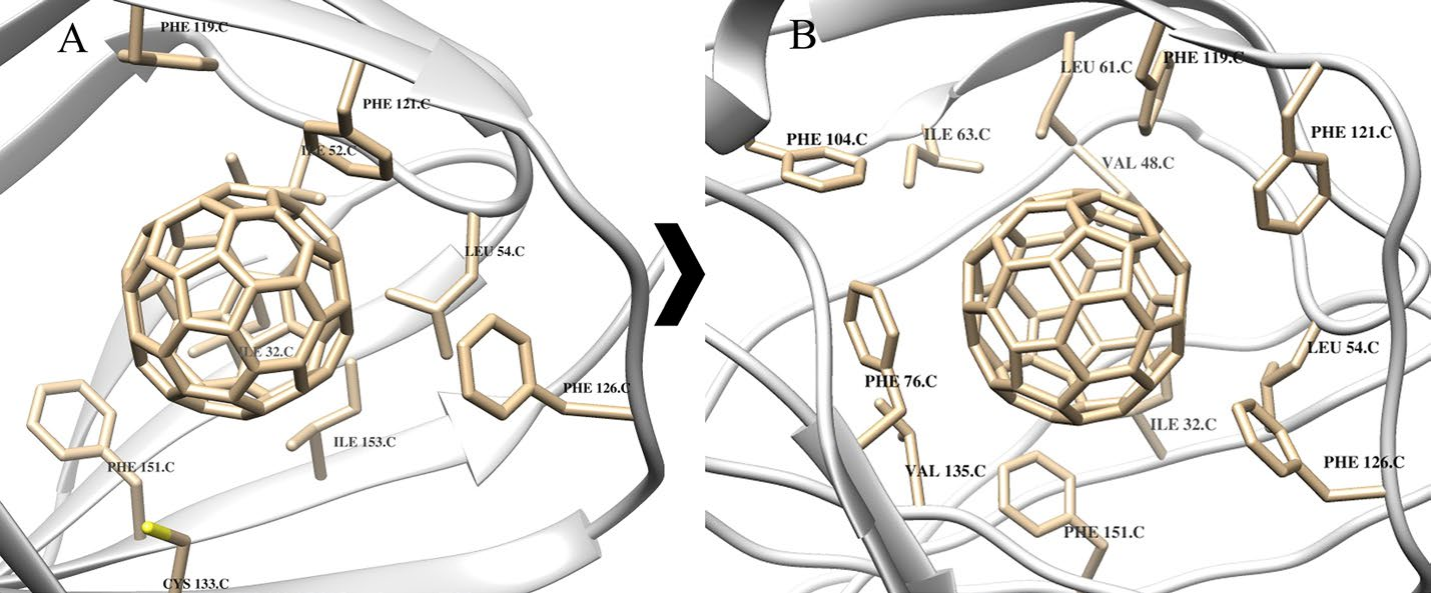
C_60_ fullerene–TLR4/MD-2-bound structure: **A** docking, **B** MD simulation

Docking and MD results showed that numerous Phe and Tyr residues easily create π–π interactions, while other amino acids (e.g., Leu, Ile, Ala, Val, and Pro) form lipophilic contacts with the sidewalls of C_60_ fullerene (Figs. 2, 3, 4, 5). In the case of binding of C_60_ fullerene with TLR1/TLR2/ECD and TLR4/MD-2, significant mobilities of protein are observed (much larger than that for the previous object): 5.2 and 4.6 Å, respectively. The obtained models were also characterized by a large energies of C_60_ fullerene–TLR complexes both after docking (free energies of complexation: − 19.2 and − 50.9 kJ/mol, respectively) and MD simulation (free energy of complexation − 26.8 and − 50.6 kJ/mol, respectively). The binding of C_60_ fullerene with TLR1/TLR2/ECDs occurs in the outer part of ECDs (Rmsd 6.7 Å) and, consequently, there is formation of new stronger stacking interactions with Phe 209 and Tyr 300 (Figs. 2, 4). The binding of C_60_ fullerene with TLR4/MD-2 is characterized by complete filling of the hydrophobic pocket of MD-2 (Figs. 3, 5) and the formation of a significant number of stacking interactions (e.g., with Phe 119, Phe 76 and Phe 104; Fig. 5B). This change of binding site is associated with significant mobility of interacting components: Rmsd value for protein is 4.6 Å, and for C_60_ fullerene—5.3 Å.

Thus, one can suggest that the formation of complexes of C_60_ fullerene, both with CYP and different parts of TLR1/TLR2 and TLR4, is potentially possible. At the same time, the calculated energy and geometrical parameters for the C_60_ fullerene–CYP complex indicate its greater stability compared with the probable C_60_ fullerene–TLR complexes.

Taking into account the results of the performed docking analysis, it was reasonable to investigate the ability of C_60_ fullerene and its nanocomplexes with anticancer drugs to modulate phagocyte metabolic processes associated with CYP and TLR signaling: ROS and reactive nitrogen species (RNS) generations, arginase activity, and phagocytosis.

### Peripheral blood phagocyte ROS generation

C_60_ fullerenes are most frequently positioned as “free radical sponges” and antioxidants due to their ability to absorb electrons (Gharbi et al. 2005; Prylutska et al. 2008). However, depending on the circumstances, C_60_ fullerenes are capable of both quenching ROS and induce their generation with or without photoexcitation (Dellinger et al. 2013; Scharff et al. 2004; Markovic and Trajkovic 2008; Prylutska et al. 2010). Moreover, spontaneous induction of ROS generation by C_60_ fullerenes plays a pivotal role in their toxic effect on eukaryotic normal and malignant cells. C_60_ fullerenes and their derivatives stimulate oxidative metabolism in erythrocytes and fibroblasts, malignant lymphocytes and epithelial cells, and endothelial cells and macrophages (Trpkovic et al. 2012). Ershova et al. (2016) reported about complex time-dependent changes in ROS levels in cells treated with C_60_ fullerene derivatives. They differentiate between “early” (from 15 min to 3 h) and “late” (24 h) responses to C_60_ fullerene exposure. Fluorescence microscopy in these experiments revealed that the early response is not associated with C_60_ fullerene penetration into the cytoplasm and is accompanied by the increase of ROS generation within the first 15–30 min on the cell surface. The late response in turn is associated with the accumulation of nanoparticles inside the cells, and includes transitory (within 1–2 h after the treatment) decrease of ROS generation followed by the secondary increase of ROS levels after the 24 h exposure.

There are two main sources of ROS in phagocytes: NADPH oxidase (NOX) and a number of cellular enzymes such as CYP and xanthine oxidase that can localize in cytosol, mitochondria, peroxisomes etc. (Dupré-Crochet et al. 2013). ROS production can be induced by the activation of TLRs 1, 2, 4 and 9. TLR-associated ROS generation primarily depends on NOX signaling pathway, and is associated with the cell surface. In addition, engagement of TLRs 1, 2 and 4 leads to the recruitment of mitochondria to phagosome followed by the activation of ROS generation in these organelles (West et al. 2011; Fagundes-Netto et al. 2013).

In our experiments, we investigated the effects of C_60_ fullerenes and their nanocomplexes with anticancer drugs Cis and Dox on intracellular ROS generation of nonsensitized human peripheral blood phagocytes in buffy coat. The treatment exposure time was 30 min. It suggests the effect of nanoformulations on ROS generation mainly through the interaction with membrane-associated receptive structures. In addition to the assessment of the effect of studied preparations on basic level of phagocyte intracellular oxidative metabolism, we also examined the so-called nonspecific reactivity reserve of the investigated function. For this purpose, cells were additionally treated with PMA that is reported to stimulate ROS generation (Campo et al. 2009). PMA activates NOX assembly in the plasma membrane, and can potentially synergize with the effect of nanoformulations (Dupré-Crochet et al. 2013). Flow cytometry method allowed us to analyze intracellular oxidative metabolism of granulocytes and monocytes separately by gating according to forward and side scatters.

**Fig. 6:**
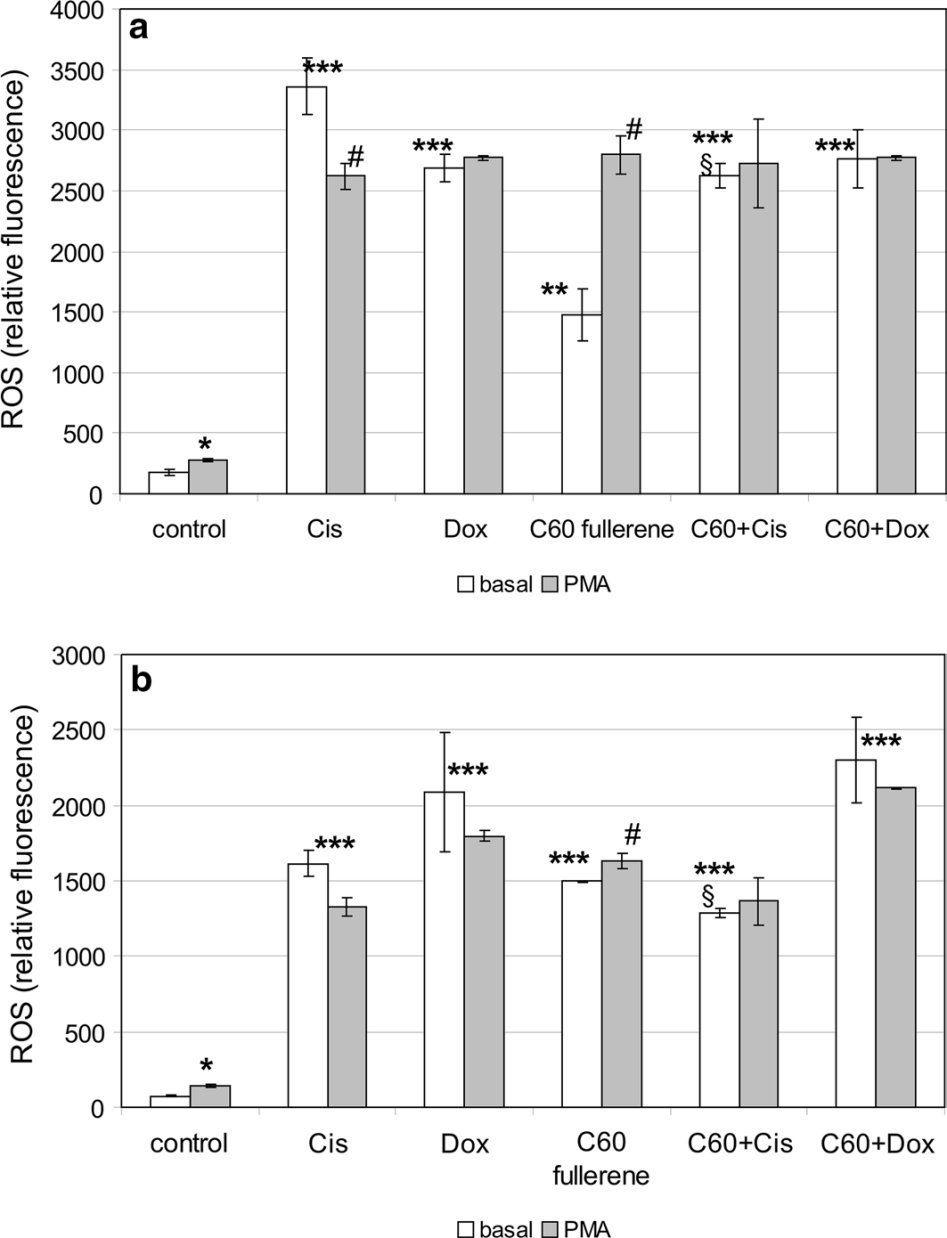
The effect of C_60_ fullerenes and their nanocomplexes with anticancer drugs Cis and Dox on intracellular ROS generation in nonsensitized human peripheral blood granulocytes (**a**) and monocytes (**b**). Whole blood samples were treated with mentioned preparations for 30 min, then were stained with carboxy-H2DCFDA (see “Methods”). The fluorescence intensities of 10.000 cells were then analyzed by BD-FACS Calibur. All results are expressed as mean ± SD of three independent experiments. *p < 0.05 compared to basal level of ROS generation in control; **p < 0.01 compared to basal level of ROS generation in control; ***p < 0.001 compared to basal level of ROS generation in control; ^#^*p* < 0.05 compared to corresponding cells without treatment with PMA; ^§^*p* < 0.05 compared to cells treated with Cis alone, as analyzed by unpaired *t* test

The treatment of peripheral blood phagocytes with all studied preparations resulted in sharp increase of intracellular ROS generation in these cells (Fig. 6). Cis and Dox caused more than 15-fold increase in the production of ROS by phagocytes. Our results are consistent with numerous literature data. Approximately half of FDA-approved anticancer drugs (including Dox and Cis) are known to produce ROS that are critically involved in toxic side effects of these drugs (Keeney et al. 2015). Reactivity reserve in cells treated with anticancer drugs in response to PMA was absent. It indicates maximal degrees of this function activation and oxidative stress development that threaten the cell viability (Fig. 6). C_60_ fullerenes caused eightfold increase in ROS generation by phagocytes. We registered the reactivity reserve in these cell samples. It indicates the less ROS-mediated toxicity of C_60_ fullerenes as compared to cytotoxic agents. Reactivity reserve of intracellular oxidative metabolism after the treatment with C_60_ fullerenes was greater in granulocytes than in monocytes as evidenced by MC values 89.7 and 25.8, respectively (Fig. 6a and b). This suggests that the granulocytes better tolerated treatment with C_60_ fullerenes. The granulocytes are terminally differentiated cells. Unlike neutrophils, circulating monocytes are less mature progenitor cells whose differentiation is completed in tissues (Dupré-Crochet et al. 2013; Rivera et al. 2016). In addition, granulocytes have only few mitochondria (Dupré-Crochet et al. 2013; Dan Dunn et al. 2015), and their contribution to the overall ROS production is minimal. Therefore, restricted mitochondria network can be another reason of better granulocyte tolerability. In mononuclear phagocytes, mitochondrial ROS generations are an important part of total intracellular oxidative metabolism. Probably, this fact along with immature state makes monocytes to be more vulnerable to the effect of C_60_ fullerenes. The complexation of Dox with C_60_ fullerene did not affect its stimulatory action on phagocyte oxidative metabolism. Whereas the complexation of Cis with C_60_ fullerene leads to the downregulation of its stimulatory effect on intracellular ROS generation.

Thus, C_60_ fullerenes both when used alone and in nanocomplexes with anticancer drugs strongly induce intracellular ROS generation in peripheral blood phagocytes, and are capable to downregulate pro-oxidant activity of Cis.

### Peripheral blood phagocyte NO production

**Fig. 7:**
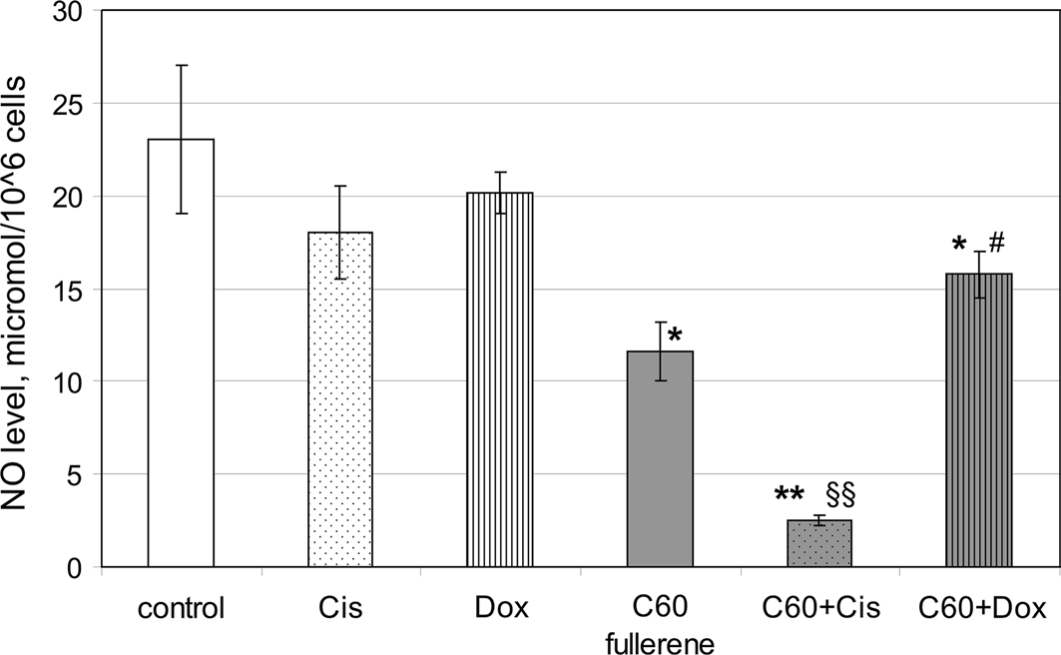
The effect of C_60_ fullerenes and their nanocomplexes with anticancer drugs Cis and Dox on RNS generation in nonsensitized human peripheral blood phagocytes. Phagocytes in buffy coat were treated with mentioned preparations for 30 min, then RNS generation was analyzed in Griess reaction (see “Methods”). All results are expressed as mean ± SD of three independent experiments. *p < 0.05 compared to control; **p < 0.01 compared to control; ^#^*p* < 0.05 compared to cells treated with Dox alone; ^§§^*p* < 0.01 compared to cells treated with Cis alone, as analyzed by unpaired *t* test

Generation of RNS is TLR-dependent metabolic process and is associated with NFκB activation (Parker et al. 2005; McCoy and O’Neill 2008). In addition, RNS synthesis is adversely regulated by oxidative stress (Singh et al. 2016) and therefore is related to CYP activation. We characterized the RNS generation by the level of nitrites (Griess reaction) in culture medium of cells treated with C_60_ fullerenes and their nanocomplexes with Cis and Dox. The Griess reaction is the most frequently used analytical approach to quantitate the major metabolites of NO, i.e., nitrite and nitrate, in a variety of biological fluids (Tsikas 2007). Nitrite production was analyzed in total pool of peripheral blood phagocytes contained in buffy coat. In our experiments, treatment of human phagocytes with Cis and Dox resulted in moderate decrease of nitrite level (Fig. 7).

Cis and Dox are reported to downregulate inducible nitric oxide synthase (iNOS) expression followed by the decrease in RNS production by affected cells. As the induction of ROS generation is a one of the mechanism of action of these anticancer drugs (Chtourou et al. 2015; Wang et al. 2016), an oxidative stress can be one of the reasons of their negative effect on RNS production. C_60_ fullerenes significantly downregulated RNS generation. Similar observations have been published by Huang et al. (2008). This research group revealed that nonfunctionalized C_60_ fullerene suppresses the release of NO by macrophages RAW 264.7. Complexation of Dox with C_60_ fullerene leads to further moderate reduction of RNS production by phagocytes. In the case of C_60_ + Cis nanocomplex, the RNS production was extremely low (by 7 times compared with cells treated with Cis alone and by 6 times compared with untreated cells). One of the probable reasons for the reduction of RNS synthesis by the treated phagocytes can be oxidative burst.

### Peripheral blood phagocyte arginase activity

**Fig. 8:**
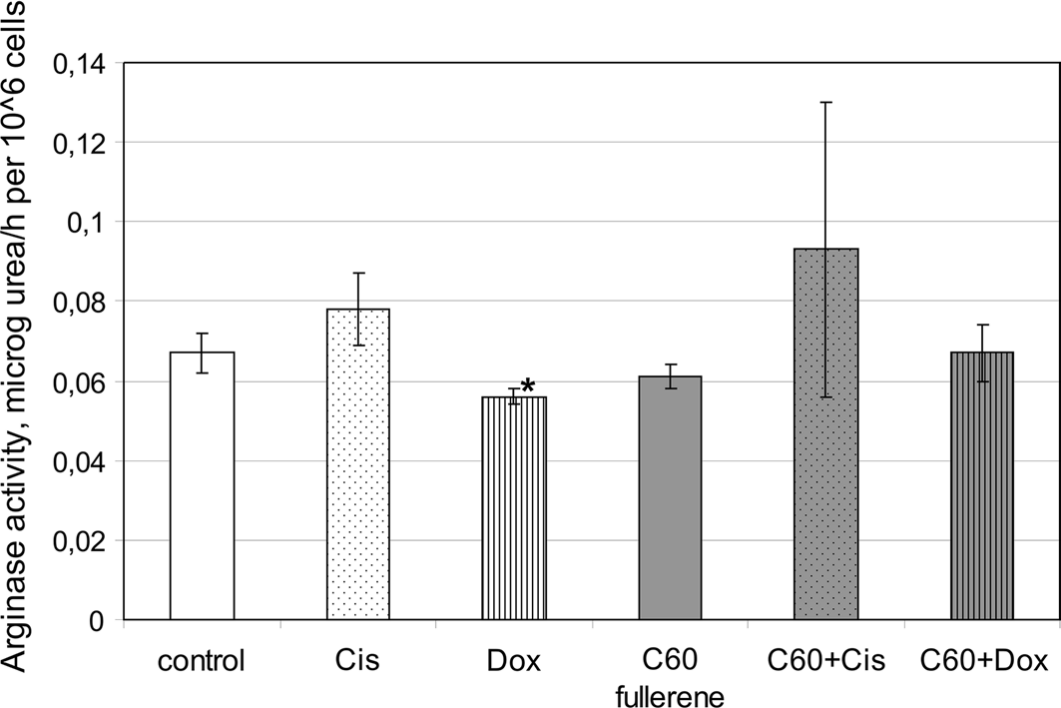
The effect of C_60_ fullerenes and their nanocomplexes with anticancer drugs Cis and Dox on arginase activity of nonsensitized human peripheral blood phagocytes. Phagocytes in buffy coat were treated with mentioned preparations for 30 min, then arginase activity was analyzed by colorimetric method (see “Methods”). All results are expressed as mean ± SD of three independent experiments. *p < 0.05 compared to control, as analyzed by unpaired *t* test

Arginase converts l-arginine into l-ornithine (precursor of proline and polyamines) and urea. iNOS and arginase can compete for the same substrate l-arginine. Overexpressed arginase can affect iNOS activity and vice versa. Increase in phagocyte arginase activity is one of the features of alternatively activated cells participating in tissue remodeling and the resolution of inflammation. Whereas the reduction of arginine metabolism through arginase activity along with iNOS activation is a sign of classical phagocyte metabolic profile, key aspect of the inflammation (Locati et al. 2013; Okabe and Medzhitov 2016; Egners et al. 2016). Arginine metabolism with arginase is associated with TLRs activation (Parker et al. 2005; Turabekova et al. 2014). The impact of C_60_ fullerene (as well as antineoplastic drugs) on arginase activity is virtually not explored. In our experiments, C_60_ fullerene when used alone did not affect this phagocyte metabolic process (Fig. 8).

Dox slightly downregulated phagocyte arginase activity. Complexation of Dox with C_60_ fullerene abrogated its inhibitory effect on phagocyte arginase activity. Cis used alone as well as in the nanocomplex with C_60_ fullerene did not affect phagocyte arginase activity. The ability of C_60_ fullerene to abrogate pro-inflammatory polarization of arginine metabolism in phagocytes caused by Dox can facilitate the reduction of cytotoxic side effect of the drugs.

### Peripheral blood phagocyte endocytosis

**Fig. 9:**
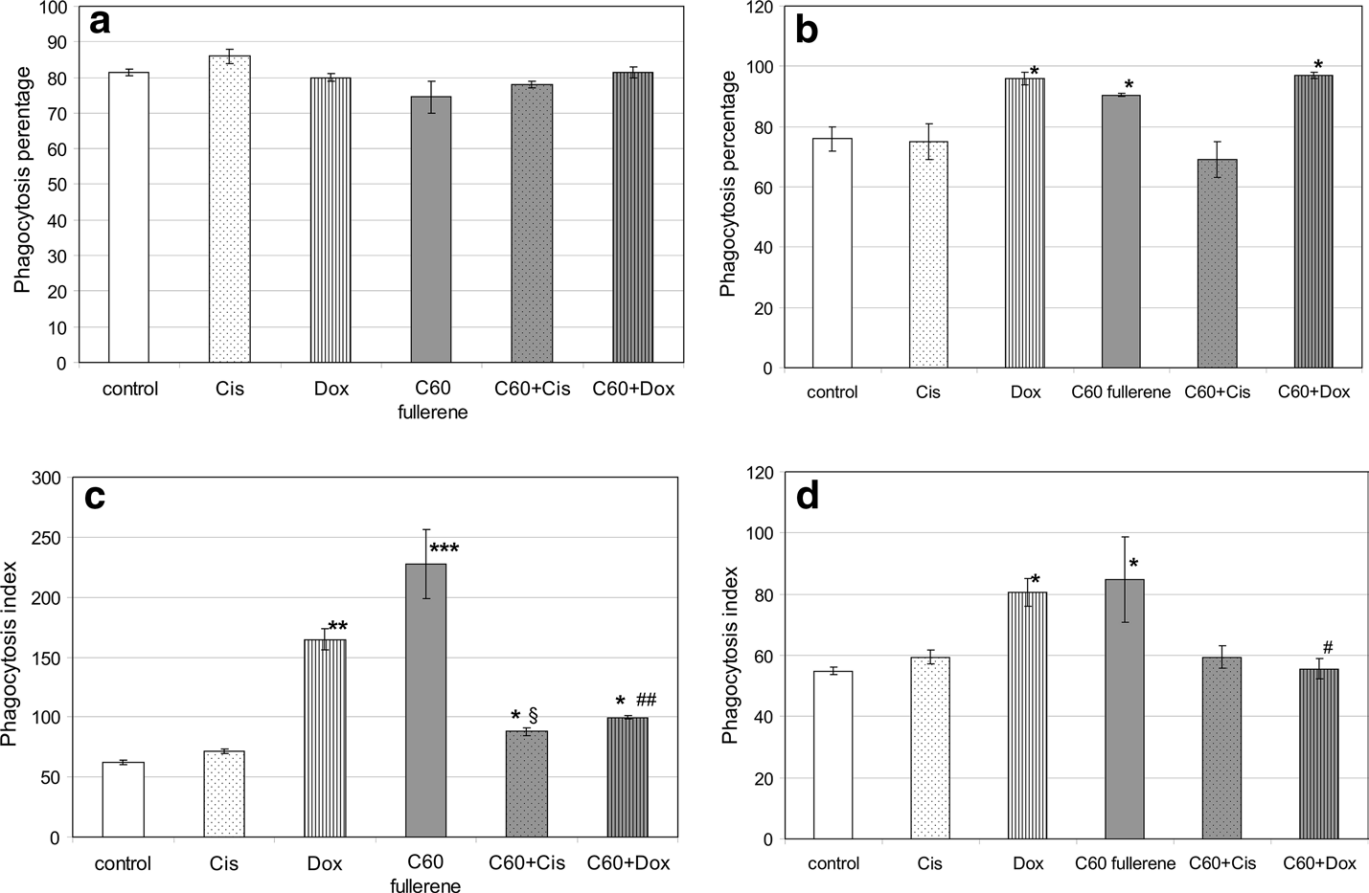
The effect of C_60_ fullerenes and their nanocomplexes with anticancer drugs Cis and Dox on phagocytic activity of nonsensitized human peripheral blood granulocytes (**a**, **c**) and monocytes (**b**, **d**). Whole blood samples were treated with mentioned preparations for 30 min, then phagocytic activity was analyzed by flow cytometry (see “Methods”). All results are expressed as mean ± SD of three independent experiments. *p < 0.05 compared to control, **p < 0.01 compared to control; ***p < 0.001 compared to control; ^#^*p* < 0.05 compared to cells treated with Dox alone; ^##^*p* < 0.01 compared to cells treated with Dox alone; ^§^*p* < 0.05 compared to cells treated with Cis alone, as analyzed by unpaired *t* test

Endocytosis (phagocytosis of solid particles and pinocytosis) is an essential part of phagocyte metabolism. Endocytosis is regulated by TLRs activation and is associated with ROS generation (Guedes et al. 2014; McCoy and O’Neill 2008; Amiel et al. 2009). The engulfment machinery differs in mononuclear (monocytes, macrophages, dendritic cells) and polymorphonuclear (neutrophils or granulocytes) phagocytes. In addition, phagocytosis plays different roles for the metabolic profile of mono- and polymorphonuclear cells. In neutrophils, phagocytosis is often associated with netosis and inflammation (Silvestre-Roig et al. 2016). In monocytes/macrophages, phagocytosis can be associated with inflammation resolution and anti-inflammatory (alternative) metabolic profile (Soehnlein and Lindbom 2010). Treatment with all investigated preparations did not affect significantly the number of phagocytizing granulocytes in tested probes (phagocytosis percentage) (Fig. 9a).

Whereas the proportion of phagocytizing monocytic cells in analyzed pool was increased after the treatment with Dox and C_60_ fullerene used alone as well as with C_60_ + Dox. All tested substance influenced phagocytosis intensity in monocytes and granulocytes. C_60_ fullerene used alone stimulated phagocyte engulfment intensity. PIs in treated phagocytes were significantly higher than that in untreated cells: 3.7 times for granulocytes and 1.6 times for monocytes (Fig. 9c and d). It is noteworthy that phagocytosis intensity in monocytes and granulocytes was characterized by significant individual variability that may result from nanoparticle size heterogeneity. Dox used alone also increased intensities of monocyte and granulocyte phagocytosis. However, its complexation with C_60_ fullerene abolished this effect. Cis used alone and in the nanocomplex with C_60_ fullerene did not affect phagocyte endocytosis intensity.

### U937 ROS generation

**Fig. 10:**
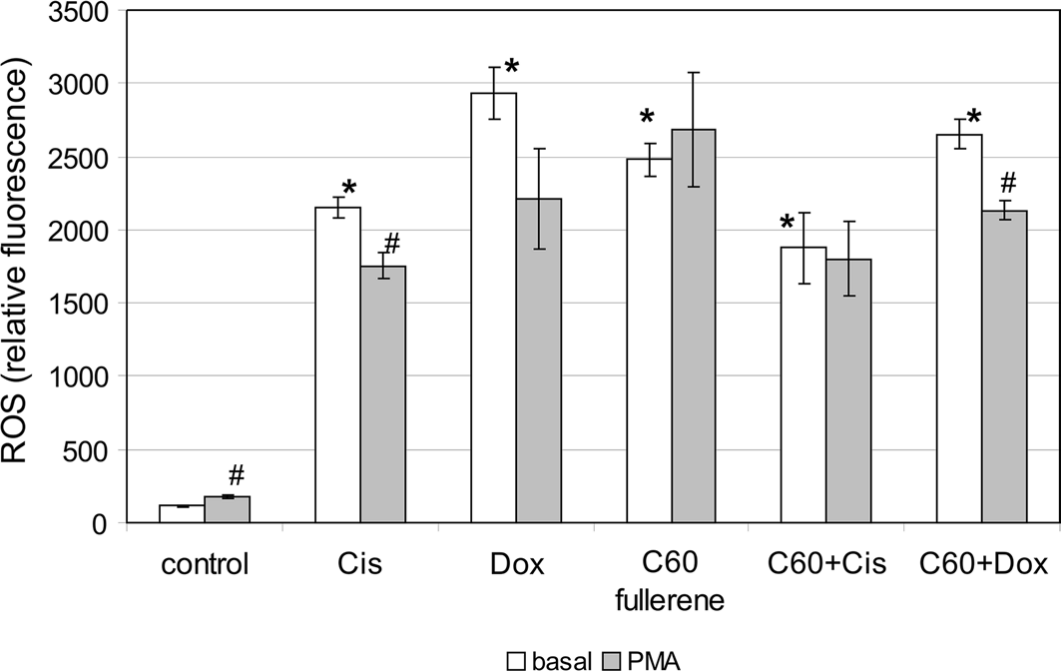
The effect of C_60_ fullerenes and their nanocomplexes with anticancer drugs Cis and Dox on intracellular ROS generation in U937 cells. U937 cells were treated with mentioned preparations for 30 min, then were stained with carboxy-H2DCFDA (see “Methods”). The fluorescence intensities of 10,000 cells were then analyzed by BD-FACS Calibur. All results are expressed as mean ± SD of three independent experiments. *p < 0.05 compared to basal level of ROS generation in control; ^#^*p* < 0.05 compared to corresponding cells without treatment with PMA, as analyzed by unpaired *t* test

As mentioned above, we observed the dramatic effect of C_60_ fullerenes and their nanocomplexes with anticancer drugs on intracellular ROS generation by peripheral blood phagocytes. Induction of ROS generation is considered as one of the mechanism of cytotoxic effect of C_60_ fullerenes (Santos et al. 2014; Zhang et al. 2015; Yu et al. 2015). Recently, activating ROS generation has become a promising approach for selective cancer treatment (Fruehauf and Meyskens 2007; Tomasetti et al. 2015). Tumor cells exhibit higher basal levels of ROS than normal cells. The intrinsic ROS stress is also characteristic for leukemic cells (Testa et al. 2016). This metabolic feature makes them more vulnerable to damage by further ROS insults induced by exogenous agents, whereas nonmalignant cells better tolerate the oxidative stress (Sun et al. 2013). It gave us the reason to estimate the effect of C_60_ fullerenes and their nanocomplexes with anticancer drugs on intracellular ROS generation phagocytic cells. To this end, we used U937 cell line. U937 is one of the most widely used myeloid cell lines (Baek et al. 2009). U937 cells of histiocytic lymphoma origin are arrested in a promonocyte/monocyte stage of differentiation (Abrink et al. 1994). A genetic analysis by Strefford et al. (2001) showed that U937 bears the *t*(10;11)(p13;q14) translocation. This results in a fusion between the MLLT10 (myeloid/lymphoid or mixed-lineage leukemia) gene and the Ap-3-like clathrin assembly protein PICALM (Clathrin assembly lymphoid myeloid leukemia), which is likely important for the tumorous nature of the cell line. Treatment exposure was 24 h. It suggests that the effect of nanoformulation could be mediated through the interaction with both membrane-associated and intracellular receptive structures (Ershova et al. 2016). Intracellular ROS levels in U937 cells, as indicated by fluorescence intensity, significantly increased in response to the treatment with C_60_ fullerenes and their nanocomplexes with anticancer drugs (Fig. 10). Oxidative stress caused by the treatment with mentioned preparations was more pronounced in transformed phagocytic cells (U937) than in normal peripheral blood phagocytes (see Fig. 6). The level of intracellular ROS in U937 cells after the treatment with C_60_ fullerenes used alone was 1.8 times higher than that in treated circulating monocytes. As shown in Fig. 6, the complexation of Cis with C_60_ fullerene caused the downregulation of its stimulatory effect on intracellular ROS production in circulating phagocytes.

Unlike, the complexation both anticancer drugs with C_60_ fullerene did not influence their pro-oxidant effect on transformed phagocytes.

Thus, C_60_ fullerenes used alone and in nanocomplexes with anticancer drugs caused dramatic increase of intracellular ROS generation in transformed phagocytes. As mentioned above, U937 cells represent the malignantly transformed myeloid cells that are arrested in a promonocyte/monocyte stage. Thus, these cells have an even more immature phenotypes in comparison with circulating normal monocytes, which turned out being more sensitive to the pro-oxidant effect of nanoformulations than neutrophils-differentiated mature cells. These results allowed us to speculate that immature state and increased basal ROS level make malignantly transformed cells more receptive to pro-oxidant effects of C_60_ fullerenes and their complexes, and potentially can be the reasons of increased cytotoxicity of nanoformulations toward transformed cells.

### Toxic effects of C_60_ fullerene and its nanocomplexes with anticancer drugs Cis and Dox on normal and malignant phagocytes

**Fig. 11:**
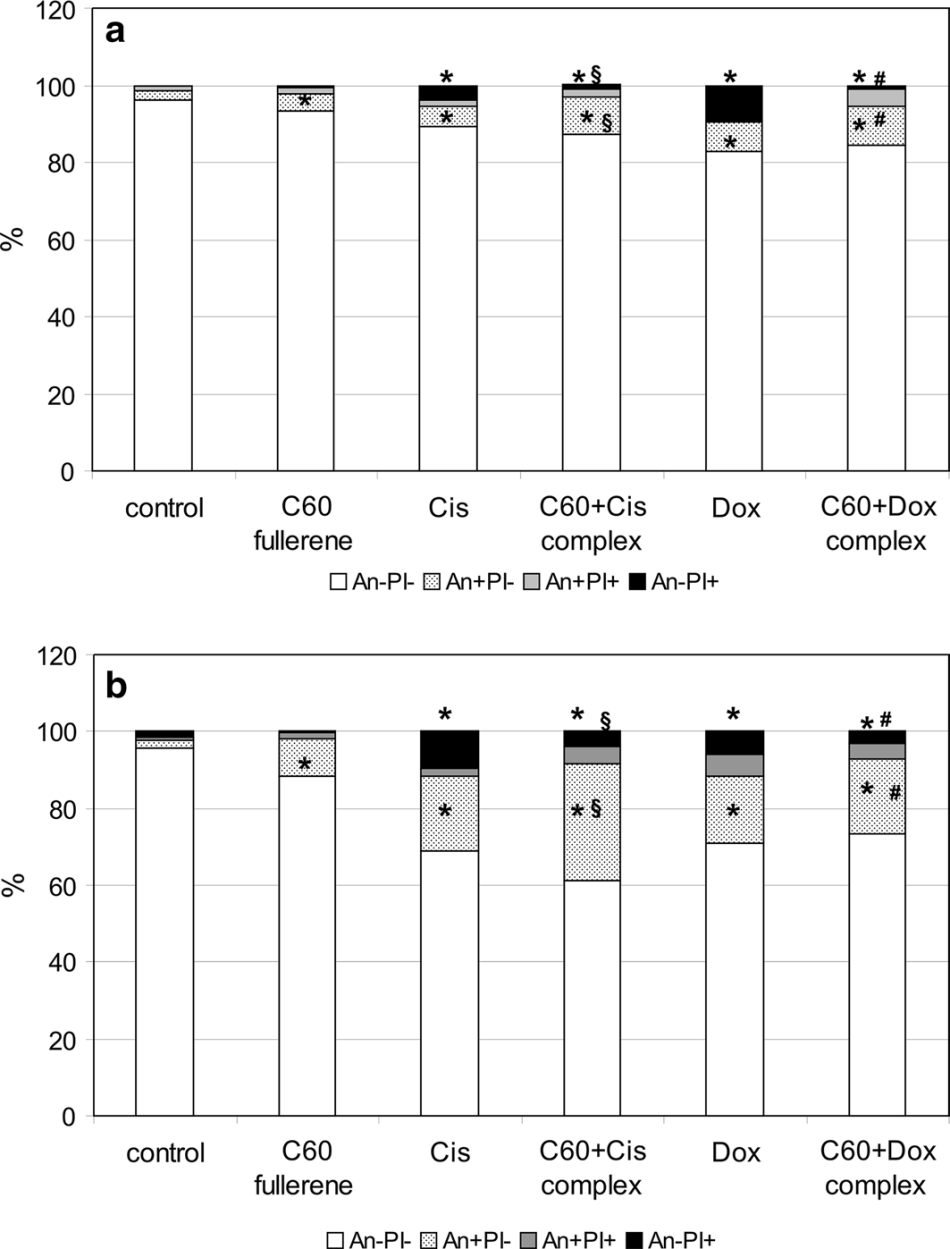
U937 cells (**b**) are more vulnerable to cytotoxic effect of C_60_ fullerenes and their nanocomplexes with Cis and Dox compared with normal monocytes (**a**). Cells were treated with mentioned compounds at the concentration of 0.15 mg/ml for 24 h. After culturing, cells were stained with annexinV (AnnV)/propidium iodide (PI) and analyzed by flow cytometry. Control: cells were incubated without any additional agents. The average values for 4 independent experiments are presented. *p < 0.05 compared with untreated cells; ^#^*p* < 0.05 compared to cells treated with Dox alone; ^§^*p* < 0.05 compared to cells treated with Cis alone, as analyzed by unpaired *t* test

To investigate the cause–effect relationship between pro-oxidant activity and cytotoxicity of C_60_ fullerenes and their nanocomplexes with anticancer drugs toward normal and transformed phagocytes, Annexin V/PI double staining of these cells treated with mentioned compounds was conducted. As shown in Fig. 11, the total number of dead normal monocytes after the treatment with C_60_ fullerenes was 6.7% vs 11.8% in the case of U937 cells. Sensitivity of transformed promonocytes/monocytes to anticancer drug-mediated death was also higher than that in normal monocytic cells. The complexation of Cis with C_60_ fullerenes resulted in slight increase in the death rate in transformed phagocytes but not in normal phagocytic cells compared with the cells treated with Cis alone. C_60_ fullerenes being used in the nanocomplex with anticancer drugs influenced the mode of cell death induced by Cis and Dox. Annexin V-FITC/PI assay allows differentiating between early apoptotic cells (An+ PI−), late apoptotic cells (An+ PI+), and necrotic cells (An− PI+). In phagocytes treated with anticancer drugs used alone, we registered higher necrosis level compared with their counterparts treated with C_60_ + Cis and C_60_ + Dox nanocomplexes. Apoptosis:necrosis ratio in cell samples treated with anticancer drugs was 2:1 (on average), whereas in the cells treated with C_60_ + Cis and C_60_ + Dox nanocomplexes, this ratio was 10:1 (on average).

## Conclusions

Thus, C_60_ fullerenes modulate phagocyte functions: stimulate phagocytic activity and downregulate RNS generation. In addition, C_60_ fullerenes can exert direct cytotoxic effect on phagocytes, more pronounced in the case of malignant cells. This cytotoxic effect is associated with the vigorous induction of intracellular ROS generation. More pronounced pro-oxidant effect of C_60_ fullerenes was also observed in transformed phagocytes. We hypothesize three main reasons for the increased receptivity of transformed cells to pro-oxidant, and thus, cytotoxic effects of nanoformulations. Two of them are the increased basal level of ROS and the immature state of transformed cells. The third reason can be the overexpression of enzymes responsible for metabolism of xenobiotics, including CYP (that is characteristic for malignant cells), if one takes into account that CYP can be considered as one of the intracellular receptive structures for C_60_ fullerenes and their nanocomplexes. On the other hand, C_60_ fullerenes have the ability to downregulate pro-oxidant effect of Cis on normal cells. Our results indicate that C_60_ fullerenes have both pro-oxidant and antioxidant properties. These results are consistent with the observations of other scientific group (Markovic and Trajkovic 2008).

## Abbreviations

ROS: reactive oxygen species
Dox: doxorubicin
Cis: cisplatin
NO: nitric oxide
MD: molecular dynamics
TLRs: Toll-like receptors
C_60_FAS: C_60_ fullerene aqueous colloid solution
